# Mitochondrial DNA and Y-chromosomal diversity in ancient populations of domestic sheep (*Ovis aries*) in Finland: comparison with contemporary sheep breeds

**DOI:** 10.1186/1297-9686-45-2

**Published:** 2013-01-22

**Authors:** Marianna Niemi, Auli Bläuer, Terhi Iso-Touru, Veronica Nyström, Janne Harjula, Jussi-Pekka Taavitsainen, Jan Storå, Kerstin Lidén, Juha Kantanen

**Affiliations:** 1 Biotechnology and Food Research, MTT Agrifood Research Finland, FI-31600, Jokioinen, Finland; 2 Department of Archaeology, University of Turku, FI-20014, Turku, Finland; 3 Department of Zoology, Stockholm University, SE-106-91, Stockholm, Sweden; 4 Archaeological Research Laboratory, Stockholm University, SE-106-91, Stockholm, Sweden; 5 Current address: Molecular Systematics Laboratory, Swedish Museum of Natural History, SE-10405, Stockholm, Sweden

## Abstract

**Background:**

Several molecular and population genetic studies have focused on the native sheep breeds of Finland. In this work, we investigated their ancestral sheep populations from Iron Age, Medieval and Post-Medieval periods by sequencing a partial mitochondrial DNA D-loop and the 5’-promoter region of the *SRY* gene. We compared the maternal (mitochondrial DNA haplotypes) and paternal (SNP *oY*1) genetic diversity of ancient sheep in Finland with modern domestic sheep populations in Europe and Asia to study temporal changes in genetic variation and affinities between ancient and modern populations.

**Results:**

A 523-bp mitochondrial DNA sequence was successfully amplified for 26 of 36 sheep ancient samples i.e. five, seven and 14 samples representative of Iron Age, Medieval and Post-Medieval sheep, respectively. Genetic diversity was analyzed within the cohorts. This ancient dataset was compared with present-day data consisting of 94 animals from 10 contemporary European breeds and with GenBank DNA sequence data to carry out a haplotype sharing analysis. Among the 18 ancient mitochondrial DNA haplotypes identified, 14 were present in the modern breeds. Ancient haplotypes were assigned to the highly divergent ovine haplogroups A and B, haplogroup B being the major lineage within the cohorts. Only two haplotypes were detected in the Iron Age samples, while the genetic diversity of the Medieval and Post-Medieval cohorts was higher. For three of the ancient DNA samples, Y-chromosome *SRY* gene sequences were amplified indicating that they originated from rams. The *SRY* gene of these three ancient ram samples contained SNP G-*oY*1, which is frequent in modern north-European sheep breeds.

**Conclusions:**

Our study did not reveal any sign of major population replacement of native sheep in Finland since the Iron Age. Variations in the availability of archaeological remains may explain differences in genetic diversity estimates and patterns within the cohorts rather than demographic events that occurred in the past. Our ancient DNA results fit well with the genetic context of domestic sheep as determined by analyses of modern north-European sheep breeds.

## Background

Archaeological and molecular genetic evidence suggests that sheep’s (*Ovis aries*) wild ancestor was the Asiatic mouflon (*O. orientalis*) and that it was domesticated about 11 000 years ago in the Fertile Crescent region [1]. The genetic history of the domestication of sheep has been investigated by analysing maternally inherited mitochondrial DNA (mtDNA) in modern sheep breeds. To date, five phylogenetically divergent mtDNA haplogroups descending probably from several *O. orientalis* populations have been identified in domestic sheep [2] i.e. haplogroups A and B that are present in sheep in many parts of the world and haplogroups C, D and E that have a much more restricted geographic range [2,3]. Sheep spread across Europe in separate migration episodes over time from their domestication site in the Near East [4]. Chessa et al. [4] have provided evidence that contemporary sheep breeds and populations of north-western and northern Europe, e.g. the Finnish native sheep breeds, Finnsheep, Kainuu Grey and Åland still share genetic ancestry with the most primitive type of sheep in Europe descending from the first immigrant wave [4].

The earliest archeological record for burned sheep bone in Finland dated by radiocarbon is from the late Stone Age (3 679 ± 33 BP, cal 2150–1950 BC; [5]). However, such archaeological evidence of animal husbandry in Finland is scarce and domesticated animals may have arrived in Finland with the expansion of the Corded Ware culture from the south via the Baltics, but also from the west (Scandinavia) and east (Russia). Available archaeological data indicate that, in Finland, sheep husbandry developed first in fairly limited areas of the southern and south-western regions. The size of the sheep population was then probably quite small and the arrival of any new animals e.g. with immigrants would have had a significant effect on the population’s gene pool. During the Iron Age, agriculture developed in eastern and central Finland until reaching the northern limit of permanent cultivation (ca. 62°N latitude) [6].

Population size, demography and morphological centeracters of medieval sheep in Finland can be inferred from historical tax registers. For example, in 1620, there were at least 188 300 sheep in Finland [7]. Foreign “Spanish” sheep (i.e. ancestors of modern Merino sheep) with finer wool were imported into Finland at least from the 16th century onwards to serve the local wool industry [8]. Exotic breeding material was introduced mainly through imported rams, which means that the admixture may not have shaped the original mtDNA diversity in the Finnish native sheep. However, nuclear marker analysis of modern sheep breeds has shown that imported animals probably had a limited effect on the gene pool of the Finnish sheep population [9]. The Finnish Sheep Breeding Organization was established in 1918 and breeding of the main native breed, Finnsheep, depended essentially on animals from the eastern part of Finland [10].

In recent years, several molecular and population genetic studies have focused on the modern Finnish native sheep breeds with analyses of within-population diversity and population structure using mtDNA, Y-chromosome markers, autosomal microsatellites and whole-genome SNP [3,9,11-15]. It has been shown that the three native Finnish sheep breeds, Finnsheep, Kainuu Grey and Åland and several other North-European native sheep breeds belong to the Nordic group of short tailed sheep [13,14]. Two different mtDNA haplogroups, A and B, segregate in the Finnish native sheep and as in other European sheep breeds; haplogroup B is much more frequent than haplogroup A [3]. In addition, Meadows et al. [12] reported that the three different Y-chromosome haplotypes assigned to two different haplogroups were present in Finnsheep, Kainuu Grey and Åland breeds.

In this study, we present a new approach to the molecular and population genetics of the Finnish native sheep breeds based on the analysis of ancient DNA (aDNA) from ancestral populations from the Late Iron Age (800–1200 AD), Medieval (1200–1550 AD) and Post-Medieval periods (1550–1800 AD). Comparing the genetic diversity of ancient populations with contemporary breeds can reveal temporal genetic changes and gene pool developments, as exemplified in a cattle Y-chromosome study [16] and in a mtDNA analysis for inferring the domestication history of European pigs [17]. However, ancient DNA studies can also reveal relatively minor changes in population diversity patterns. For example, frequencies of the mtDNA haplogroups A and B detected in ancient Chinese sheep populations from the Bronze Age are similar to those in contemporary Chinese sheep breeds [18,19].

We investigated the genetic diversity of ancient sheep remains in Finland by sequencing a 523-bp mtDNA D-loop sequence and a 130-bp segment in the 5’-promoter region of the ovine *sex determining region Y* (*SRY*) gene to detect a biallelic Y-chromosome SNP marker *oY*1. We compared mtDNA and Y-chromosome marker data from ancient and modern domestic sheep populations in Finland and other parts of Europe and Asia and studied temporal changes in maternal and paternal genetic diversity and mtDNA diversity patterns. For the mtDNA study, two modern datasets were used: (1) 10 modern European sheep breeds were sequenced and (2) additional mtDNA sequences were collected from GenBank that spanned the same mtDNA D-loop nucleotide sites as those present in the ancient mtDNA sequences. To our knowledge, this is the first time that mtDNA and Y-chromosome genetic diversities of ancient sheep populations are investigated in the same study.

## Materials and methods

### Ancient sheep bone material

For the aDNA analysis, 36 sheep bones were selected from 14 different locations and 18 different archaeological excavations across Finland and one site in northern Norway (Table 1, Additional file 1: Figure S1). As a rule, unburned bone from time periods prior to the Late Iron Age is not preserved in the Finnish acid soil; thus earlier bone material consists of small burned fragments that are not useful for aDNA analyses [20]. The earliest unburned bones available for this study derive from the Late Iron Age (800–1200 AD). 

**Table 1:** Summary of ancient samples studied in this article

**Sample**				**Dating**		**aDNA**	
**Sample ID**	**Location**	**Site**	**Bone type**	*BP (± 1σ)	**Period	**mtDNA**	***SRY*gene**
OaMik1	Mikkeli	Moisio Latokartano [21]	Mandible	865 ± 33	Iron Age	No	
OaM1	Raisio	Mulli [22]	Metacarpal	965 ± 30	Iron Age	Yes	unreadable
OaM2	Raisio	Mulli [22]	Metacarpal	1040 ± 31	Iron Age	Yes	No
OaM3	Raisio	Mulli [22]	Metacarpal	955 ± 30	Iron Age	Yes	unreadable
OaM4	Raisio	Mulli [22]	Metacarpal	995 ± 30	Iron Age	Yes	unreadable
OaM5	Raisio	Mulli [22]	Metacarpal	1081 ± 32	Iron Age	Yes	unreadable
OaSys1	Sysmä	Ihananiemi [23]	Tooth (Molar)	1093 ± 31	Iron Age	No	
OaKir1	Turku	Kirkkomäki [24]	Tooth fragments	NA	Iron Age	No	
OaLui1	Eura	Luistari [25,26]	Tooth fragments	NA	Iron Age	No	
OaLui2	Eura	Luistari [25,26]	Tooth fragments	NA	Iron Age	No	
OaPas1	Pasvik (Norway)	Brodtkorbneset [27]	Metatarsal	984 ± 31	Iron Age	Partial	No
OaNaa1	Naantali	Luostari [28]	Metacarpal	452 ± 30	Medieval	Yes	Yes
OaUuk2	Uukuniemi	Papinniemi [29]	Metatarsal	410 ± 30	Medieval	Yes	No
OaÅA3	Turku	Åbo Akademi [30]	Metacarpal	506 ± 32	Medieval	Yes	No
OaÅA4	Turku	Åbo Akademi [30]	Metacarpal	581 ± 31	Medieval	Yes	No
OaÅA5	Turku	Åbo Akademi [30]	Metacarpal	722 ± 32	Medieval	Yes	No
OaÅA6	Turku	Åbo Akademi [30]	Metacarpal	737 ± 32	Medieval	Yes	No
OaVet1	Turku	Aboa Vetus [28,31]	Horncore	487 ± 30	Medieval	No	
OaVet2	Turku	Aboa Vetus [28,31]	Maxilla	DBC	Medieval	No	
OaVet3	Turku	Aboa Vetus [28,31]	Axis	550 ± 30	Medieval	No	
OaVet4	Turku	Aboa Vetus [28,31]	Lower jaw	DBC	Medieval	No	
OaKök1	Kökar	Kloster [32]	Metacarpal	489 ± 30	Medieval	Yes	No
OaHel1	Helsinki	Snellmaninkatu [33]	Metatarsal	DBC	Post-Med	Yes	No
OaHel2	Helsinki	Snellmaninkatu [33]	Metatarsal	DBC	Post-Med	Yes	No
OaOul1	Oulu	Kajaaninkatu [34]	Radius	DBC	Post-Med	Yes	No
OaOul2	Oulu	Lyseo [35]	Radius	DBC	Post-Med	Yes	No
OaOul3	Oulu	Pikisaari [36]	Radius	DBC	Post-Med	Yes	No
OaPie1	Pietarsaari	Lassfolk [37]	Metacarpal	DBC	Post-Med	Yes	No
OaTor1	Tornio	Keskikatu [38]	Metacarpal	DBC	Post-Med	Yes	Yes
OaTor2	Tornio	Keskikatu [38]	Metacarpal	DBC	Post-Med	Yes	No
OaÅA1	Turku	Åbo Akademi [30]	Metacarpal	DBC	Post-Med	Yes	unreadable
OaÅA2	Turku	Åbo Akademi [30]	Metacarpal	DBC	Post-Med	Yes	No
OaPih1	Pihtipudas	Hämeensaari [39]	Tibia	342 ± 30	Post-Med	Yes	No
OaKök2	Kökar	Kloster [32]	Metacarpal	305 ± 30	Post-Med	Yes	Yes
OaKök3	Kökar	Kloster [32]	Metacarpal	DBC	Post-Med	Yes	No
OaKök4	Kökar	Kloster [32]	Metacarpal	DBC	Post-Med	Yes	No

Identity of samples include: sample ID, location i.e. town where samples were excavated, archaeological site, and type of bone (museums ID available upon request); dating of samples include radiocarbon-dates (*BP (± 1σ)) or dates estimated by the context (DBC) and corresponding historical **periods (Post-Med = Post-Medieval); results of mtDNA D-loop and Y-chromosomal *SRY* gene sequence analysis are indicated for each sample; only the samples yielding mtDNA were analysed for the *SRY* gene.

When possible, metacarpal or metatarsal bones were selected for two reasons: (1) these bones are easy to distinguish between sheep and goats and (2) they are often found in a complete or semi-complete state in the excavations because since they are nutritionally poor they were not usually butchered for cooking. However, other bone elements were also sampled, e.g. when metapodials were not present in the sample, or were badly preserved or fragmented, or if they did not form the highest Minimum Number of Individuals (MNI) within the excavation or phase. In some cases, only bones that are directly identifiable as from sheep or goat (e.g. teeth) were selected because it is possible to identify a species from analyses of the mtDNA D-loop region. Care was taken not to sample the same individual twice i.e. within one site and phase, elements located on the same side were selected for a given sample, otherwise age and size of the animal were used to separate different individuals. All samples that based on the archaeological context were assumed to originate from the Iron Age or Medieval period were radiocarbon-dated. In addition, Post-Medieval samples for which dating was uncertain from the archaeological context, were also radiocarbon-dated. Nineteen sheep bones or teeth were radiocarbon-dated in the Laboratory of Chronology of the Finnish Museum of Natural History, University of Helsinki (Table 1).

#### Iron age

Eleven samples excavated at four different sites from the Iron Age were included in this study (Table 1, Additional file 1: Figure S1). The aDNA samples (sample ID: OaM1-5) belong to the Viking Age phase of the Mulli site (1090–930 BP, Table 1). The animal bone assemblage from this site is mainly composed of domestic animals, but also contains a variety of wild mammals. Eastern Finland Viking Age sites were sampled (OaSys1 and OaMik1) and samples from two Iron Age cemetery sites in south-west and southern Finland (OaLui1-2 and OaKir1) were collected. The sample from Brodtkorbneset, Pasvik (Norway) (OaPas1) derives from a rectangular hearth of Sami origin and also dates to the late Iron Age (984 ± 31 BP, Table 1).

#### Medieval period

Eleven samples from western and eastern Finland from the Medieval period were analysed (Table 1, Additional file 1: Figure S1) among which eight originated from the town of Turku (OaÅA3-6, OaVet1-4), the largest Medieval town in Finland that is situated on the south-west coast by the river Aura, one from Bridgettine Abbey of Naantali (OaNaa1) situated in south-west Finland ca. 18 km from Turku, one from Kökar monastery, a medieval Franciscan monastery situated in the Åland archipelago (OaKök1), and one from eastern Finnish Karelia i.e. a deserted Greek Orthodox village in Papinniemi in Uukuniemi (OaUuk2).

#### Post-Medieval period

Fourteen samples from the Post-Medieval period were analysed (Table 1, Additional file 1: Figure S1) among which six were collected from the shores of the Gulf of Bothnia, from Pietarsaari (OaPie1), Tornio (OaTor1-2) and Oulu (OaOul1-3), seven from south-west and southern Finland i.e. two from Kökar monastery (OaKök2-4), two from Turku (OaÅA1-2) and two from Helsinki (OaHel1-2), both in urban contexts and one from an inland region at Pihtipudas (OaPih1).

### Modern sheep

The occurrence of ancient sheep mtDNA sequences in modern sheep breeds was investigated with two datasets: (1) our own dataset of 94 unrelated animals from 10 sheep breeds or local varieties [see Additional file 1: Figure S1] and (2) a dataset composed of GenBank sequence data of 50 European and Asian sheep breeds previously published. Our own dataset was also used for the comparison between ancient and modern DNA. Previously described in [3,14], breeds included in our own dataset are the following: the Finnish native sheep breeds Finnsheep, Kainuu Grey and Åland, the Viena sheep from Russian Karelia, Bozakh sheep from the Caucasus, Romanov and Oparino sheep from central Russia, Olkuska sheep from Poland, the morphological type of Vlashko Vitoroga–Pramenka sheep from Serbia and Oxford Down sheep from the UK. All sheep breeds were locally developed breeds except for Oxford Down, which is a synthetic commercial English breed [40]. Kainuu Grey, Åland, Oparino, Olkuska and Vlashko Vitoroga-Pramenka sheep have undergone a reduction in population size during the last 10 years and are rare or endangered breeds. Results from a Y-chromosome SNP *oY*1 analysis in ancient sheep were compared with those of a global sheep Y-chromosome study including Finnsheep, Kainuu Grey and Åland sheep [12].

### DNA extraction and laboratory methods

#### Laboratory environment and DNA extraction of ancient sheep samples

Bone samples were prepared by removing the outer layer of the bones and collecting 50 to 200 mg of bone powder with a drill. DNA extraction was carried out in an air-controlled sterile laboratory and in a laminar flow hood (EU-14 HEPA filtered air under positive air pressure isolation). Separate laboratories were used for sample preparation, DNA extraction and PCR amplification. The laboratories and equipment were UV-treated and bleach was used to clean the laminar and laboratory regularly. Protective whole body suits, double gloves and masks were used inside the aDNA laboratory. Two aDNA laboratories participated in aDNA analyses (MTT Agrifood Research Finland and Stockholm University, Sweden).

DNA extraction was carried out as described in [41]. The bone powder was digested in 900 μL 0.5 M EDTA, 100 μL 10 M urea and 5 μL proteinase K (20 mg/ml) with constant stirring at 55°C overnight. After centrifugation (2000 rpm for 5 min), the supernatant was concentrated and DNA was extracted with a QIAquick PCR Purification Kit (Qiagen, Sweden) according to the manufacturer’s instructions.

#### DNA markers, primer design and PCR

A 523-bp sequence of mtDNA encompassing part of the mtDNA D-loop from ancient sheep ([GenBank:NC001941] positions 15,978-16,501) was analysed. A 130-bp sequence in the 5’-promoter region of the *SRY* gene on ovine Y-chromosome ([GenBank:AY604734] positions 58–179) was analysed to detect the Y-chromosome SNP marker (*oY*1) [GenBank:AY604734.2:g.67, A>G].

Primers [see Additional file 2: Table S1] for five overlapping fragments of mtDNA D-loop sequence and one Y-chromosome *SRY* gene fragment were designed with Primer3 [42] using mtDNA and *SRY* gene reference sequences [GenBank:NC001941] and [GenBank:AF026566.1], respectively.

PCR for ancient DNA was performed in 25 μL mixture that contained 1× PCR buffer (Qiagen, Sweden), 0.2 μM of each primer, 0.4 mM dNTP, 2.5 mM MgCl_2_ (Qiagen, Sweden), 0.25 units (U) of Uracil DNA Glycosylase (UNG, Sigma-Aldrich), 1.5 U of HotStarTaq DNA Polymerase (Qiagen, Sweden) and 5–10 μL of DNA extract. The PCR program consisted in initial steps at 37°C for 10 min and 95°C for 15 min followed by 55 three-step cycles at 94°C for 30 s, AT°C for 40s and 72°C for 1 min and a final step at 72°C for 10 min. AT stands for the annealing temperature specific to each primer set [see Additional file 2: Table S1].

### Authenticity of ancient sheep DNA

Common measures to prevent contamination were used [43,44], such as separated areas for sample preparation, ancient DNA analyses and pre-PCR, wearing protective clothing, using disposable tools and pipettes with aerosol resistant filter tips and treating equipment and working surfaces with bleach and ultra-violet irradiation frequently (see DNA extraction and laboratory methods). Overlapping primers specific to sheep DNA were designed to prevent possible annealing to human DNA and were checked by amplification (see DNA extraction and laboratory methods).

The authenticity of the aDNA analysis was controlled at various steps of the laboratory work-flow. In general, when consistent sequences were obtained in three or more amplifications, the sequence was considered reproducible and accepted as authentic. In the aDNA extraction step, 13 of the samples (out of the 26 samples which were successfully amplified for the 523-bp mtDNA D-loop sequence) (Table 1) were extracted several times, and at least two PCR-reactions were performed for separate DNA extractions to amplify and sequence each overlapping mtDNA D-loop fragment at least twice. Applying these strict extraction and amplification steps aimed at confirming the reproducibility of our aDNA sequence protocol and no sequence anomalies were detected. Moreover, five samples (OaM1, OaNaa1, OaTor2, OaÅA5 and OaÅA6) were extracted, amplified and sequenced in two different aDNA laboratories (MTT Agrifood Research Finland and Stockholm University, Sweden) and the results were identical. At each step of the aDNA extraction and amplification procedure, negative controls were performed. In the first extraction of our aDNA trial, a mammoth sample was used as positive control, which constitutes a suitable control because a previous study [45] has shown that it does contain mammoth DNA, it does not contain modern DNA and its sequence clearly differs from that of sheep.

### Mitochondrial DNA amplification of modern sheep samples

DNA from 94 modern sheep of 10 European and Asian breeds [3,14] were analysed. Additional file 2: Table S1 includes primers for the amplification of a 664-bp sequence of mtDNA [see Additional file 2: Table S1]. The same PCR reaction mix was used for these samples than for the aDNA samples except that UNG was not included. PCR conditions were as follows: 95°C for 15 min followed by 32 three-step cycles at 94°C for 30 s, 58°C for 40 s and 72° for 1 min and a final step at 72°C for 10 min. 500 ng of template DNA were used per reaction. Y-chromosome marker data were available from a previous study [12].

### Sequencing of PCR products

PCR products of modern and ancient DNA samples were purified using ExoSAP-IT enzyme (GE Healthcare Life sciences, UK). Sequencing reactions were performed using DYEnamic ET Terminator Kit (GE Healthcare Life sciences, UK). The sequencing products were purified by ethanol precipitation and separated on MegaBACE1000™ (Amersham Biosciences, UK). Both strands of each fragment were sequenced and the same primers were used for both sequencing and fragment amplification. Sequence data were base-called with Cimarron 3.12 basecaller using a MegaBACE Sequence analyzer v. 3.0.0111.1603 (Amersham Biosciences, UK). Sequences were analysed with Sequencher 4.6 8 (Gene Codes, Ann Arbor, MI).

### Statistical analyses

Mitochondrial DNA sequences from 94 modern sheep [GenBank: JX484017-JX484110] and 26 ancient sheep [GenBank: JX484111-JX484137] were aligned using CLUSTALW2 [46] (penalties for gap opening, gap extension, gap distances were 10, 0.20 and 5, respectively). When statistical analyses were performed for a subset of sequences, the CLUSTALW2 alignment was carried out separately for each cohort. The size of the aligned mtDNA sequence was 523-bp. One Iron Age sample (OaPas1), for which only 297-bp of the mtDNA D-loop was sequenced, was omitted from the statistical analyses (Table 1). A corresponding CLUSTALW2 alignment was done for the Y-chromosome 130-bp sequences [JX484138- JX484140]. Alignment gaps were excluded from the statistical analyses.

The appropriate DNA substitution model to analyse our mtDNA data was the Hasegawa-Kishino-Yano model ([47]; HKY85)+Г supported both by FindModel [48] web server (http://www.hiv.lanl.gov/content/sequence/findmodel/findmodel.html) and MEGA5.05 program [49] and selected on the basis of the Akaike information criterion (AIC) [48] for different models. The phylogenetic analysis of the 94 modern and 26 ancient sheep sequences and one outgroup sequence from urial sheep (*Ovis vignei bocenteriensis* [GenBank:AF039580.1]) was conducted using two approaches. MEGA5.05 was used to construct the Neighbor-joining (NJ) tree with 1000 bootstrap replicates. However, the Tamura-Nei substitution model [50] with a Г distribution parameter value α = 0.05 was used in the analysis because HKY is not implemented in the MEGA software and the Tamura-Nei model was supported by AIC. The maximum likelihood (ML) analysis was performed using PhyML v. 3.0 program [51] and the HKY85+Г (lnL = −1271.48434, Г distribution parameter value α = 0.047). Bootstrap support for a ML tree was calculated using 1000 bootstrap replicates and the tree was drawn with the TreeView program v. [52]. In addition, a median-joining network (with ε = 0 to be most conservative) between the haplotypes was constructed and mismatch distribution was performed using NETWORK 4.6.0.0 [53].

The following parameters were calculated to estimate the genetic diversity of the mtDNA data in the different cohorts (i.e., in the ancient populations and modern breeds): number of haplotypes (h), number of segregating sites (S), haplotype diversity (Hd = probability that two mtDNA sequences chosen randomly from the sample are different), nucleotide diversity (π = number of nucleotide differences between randomly chosen pairs of sequences), and average number of nucleotide differences (K) (DnaSP v.5 [54]). Theta-estimates (‘theta’ θ = N_e_μ, where N_e_ is the effective population size in the case of a haploid locus and μ is the overall mutation rate at the haplotype level) were computed using ARLEQUIN v. 3.5 [55]: the expected level of diversity, θ_S,_ was derived from the observed number of segregating sites S and θ_π_ from the observed mean number of pairwise nucleotide differences π. Furthermore, Tajima’s D test statistic was computed using DnaSP.

We investigated the distribution of the ancient mtDNA sequences present among the 94 sequences of the modern sheep samples sequenced here and searched for shared identical sequences in the GenBank DNA database (NCBI/BLAST, http://blast.ncbi.nlm.nih.gov/Blast.cgi). The haplotypes were determined using DnaSP [see Additional file 3: Table S2].

The ancient Y-chromosome SNP *oY*1 data were compared with modern data [9,12] and geographic frequency distributions of *oY*1 alleles. The modern Y-chromosome SNP data [9,12] on the frequencies of *oY*1 alleles are presented in Additional file 4: Table S3.

## Results

### Radiocarbon dating

Nineteen samples were successfully radiocarbon-dated, while for three samples, the quantity of collagen in the enamel was not sufficient for this technique (Table 1). The radiocarbon dating indicated that two samples, (OaPih1, OaKök2), were younger and one (OaUuk2) was older than inferred from the archaeological records from the same sites (Table 1). The dating results indicated that the oldest samples successfully analyzed for mtDNA were approximately 1000 years old.

### Success rate of aDNA analyses

Mitochondrial DNA analysis was carried out on 36 ancient sheep samples and mtDNA amplification was successful for 27 samples, including the partial mtDNA sequence of the OaPas1 sample (Table 1). Most of these 27 samples required several amplifications per fragment in order to obtain at least three good-quality sequences from at least two PCR reactions. Excluding the samples for which no mtDNA amplification was obtained and considering all separate amplifications for the five mtDNA D-loop fragments, the amplification success rate was 46%, 56% and 68% for sheep samples from the Iron Age, Medieval and Post-Medieval periods, respectively. Average amplification success rates and fragment lengths for the mtDNA D-loop sequence are summarised in [Additional file 2: Table S1]. As expected, with aDNA samples, the highest success rate was obtained when the amplified fragments of mtDNA D-loop were shortest. In addition, among the aDNA samples from different periods, those from the Post-Medieval period had the highest success rate, which is explained by the fact that Post-Medieval material is abundant allowing a more critical prior-selection of bones than for older materials. Reproducible sequences were obtained for seven of the 11 Medieval samples and for five (plus one partial) of the 11 Iron Age samples (Table 1).

Amplification of a 130-bp sequence of Y-chromosome 5’ promoter region in the *SRY* gene was tested in aDNA samples that were successfully amplified for mtDNA and three contained the *SRY* gene sequence. As expected, among the different aDNA analyses, amplification success rate was lowest (12%) for the fragment containing the *SRY* gene.

### Mitochondrial DNA haplotypes

**Figure 1:**
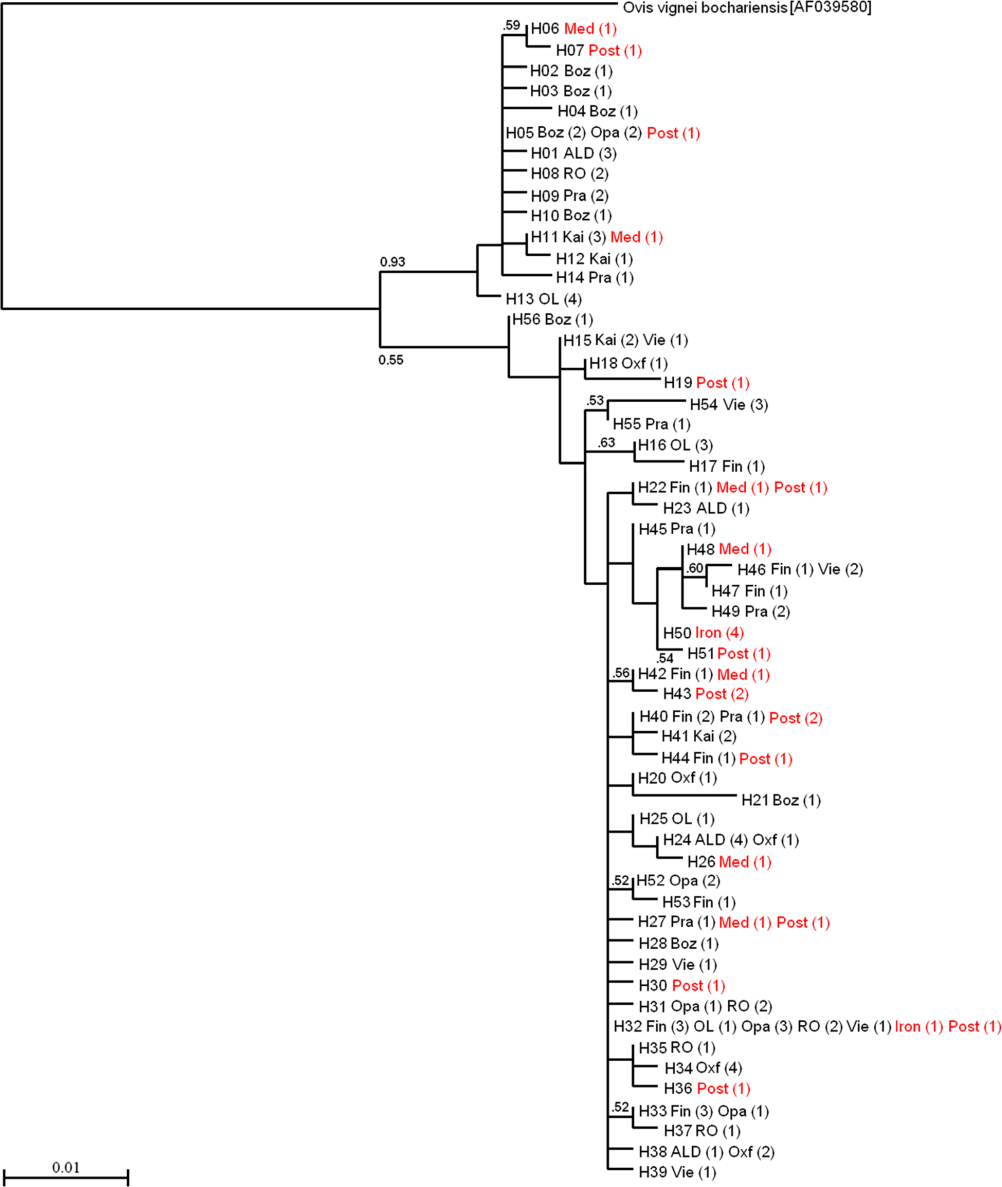
**Maximum Likelihood tree of mtDNA haplotype sequences found within modern (black) and ancient (red) sheep.** Branch topography supported by bootstrap values greater than 50% is indicated; the number of samples sharing haplotypes is given in brackets; breed names are abbreviated as Oxford Down (Oxf), Olkuska (OL), Pramenka (Pra), Bozakh (Boz), Oparino (Opa), Romanov (Ro), Viena (Vie), Kainuu Grey Sheep (Kai), Åland (ALD) and Finnsheep (Fin); ancient Finnish sheep samples are divided into three periods: Iron Age (Iron), Medieval (Med) and Post-Medieval (Post) according to radiocarbon or context dating (see Table 1); the tree is rooted with a sequence from urial sheep (*Ovis vignei bocenteriensis*) [GenBank: AF039580.1].

Alignment between the 94 modern and 26 ancient DNA sequences (OaPas1 containing a partial mtDNA sequence was excluded) revealed 47 SNP (46 transitions and one transversion) and three insertion-deletions [see Additional file 5: Figure S2]. When considering the alignment of the aDNA samples only, 27 transitions and no transversion were found. Fifty-six haplotypes (18 in the ancient and 46 in the modern DNA sequences) were identified among the 121 modern and ancient sheep samples (Figure 1, Tables 2 and 3). More than half of these haplotypes (30 of 56) were private to one individual and thus to one modern sheep breed or ancient sheep population indicating high variation in the analysed D-loop region.

**Table 2:** Summary statistics of ancient Finnish sheep populations from three periods and modern Finnish sheep breeds

**Statistics**	**Ancient Finnish sheep**			**Modern Finnish sheep breeds**		
	**Post-Medieval**	**Medieval**	**Iron Age**	**Åland**	**Finnsheep**	**Kainuu Grey**
N	14	7	5	9	15	8
S	25	18	2	17	14	15
h	12	7	2	4	10	4
Hd	0.98	1.00	0.40	0.75	0.93	0.82
K	6.13	7.81	0.80	7.94	2.61	8.11
π	11.72	14.93	1.53	15.19	4.99	15.50
D	−0.934	0.352	−0.973	1.315	−1.562	2.054*
θs	7.861	7.347	0.960	6.255	4.306	5.785
θπ	6.396	8.095	0.800	7.944	2.743	8.107
θπ-θs	−1.465	0.748	−0.16	1.689	−1.563	2.322

N, number of sampled individuals; S, number of segregating sites (excluding indels); h, number of haplotypes; Hd, haplotype diversity; K, average number of differences; π, nucleotide diversity*10^-3^; D, Tajima´s D statistic value where statistically significances P < 0.05 are marked with *; θs, ‘Theta’ derived from the observed number of segregating sites (S) and θ_π_ from the observed mean number of pairwise nucleotide differences (π).

**Table 3:** Summary statistics of modern Polish, Russian and UK breeds

**Statistics**	**Modern sheep breeds**						
	**Bozakh**	**Olkuska**	**Oparino**	**Oxford Down**	**Pramenka**	**Romanov**	**Viena**
N	9	9	9	9	9	8	9
S	21	14	16	9	19	16	11
h	8	4	5	5	7	5	6
Hd	0.97	0.75	0.86	0.81	0.94	0.89	0.89
K	7.44	7.39	5.89	2.83	8.11	6.96	4.39
π	14.23	14.13	11.26	5.42	15.51	13.32	8.39
D	−0.180	2.085*	0.002	−0.664	0.786	0.661	0.397
θs	7.727	5.151	5.887	3.311	6.991	6.171	4.047
θπ	7.667	7.389	5.889	2.833	8.333	6.964	4.389
θπ-θs	−0.06	2.238	0.002	−0.478	1.342	0.793	0.342

N, number of individuals sampled; S, number of segregating sites (excluding indels); h, number of haplotypes; Hd, haplotype diversity; K, average number of differences; π, nucleotide diversity*10^-3^; D, Tajima´s D statistic value where statistically significances P < 0.05 are marked with *; θs, ‘Theta’ derived from the observed number of segregating sites S and θ_π_ from the observed mean number of pairwise nucleotide differences π.

The NJ and ML analyses gave similar phylogenetic topologies thus only the ML tree is presented in Figure 1. Two highly divergent domestic sheep lineages, ovine mtDNA haplogroups A and B, were detected with relatively high statistical support. As expected, comparisons with reference sequences [GenBank: AF039577.1] and [GenBank: AF039578.1], haplogroups B and A were respectively the major and minor haplogroups. Frequencies of haplogroups A and B were 0% and 100% for the Iron Age, 28.6% and 71.4% for the Medieval and 14.3% and 85.7% for the Post-Medieval sheep cohorts, respectively and 21.9% and 78.1% for the Finnish modern sheep breeds. In addition, the Network analysis and mismatch distribution supported the existence of these two divergent haplogroups in the modern sheep breed data presented in Figures S3 and S4 [see Additional file 6: Figures S3 and S4]. These analyses reproduced the well-established observations with a star-shaped pattern in the ovine mtDNA haplotype network and a smooth shape of mismatch distribution indicating a population expansion in the history of the species. Similar patterns were observed in the Medieval-Iron Age and Post-Medieval cohorts when analysed separately (results not shown).

We analysed the distribution of the 18 ancient Finnish sheep mtDNA haplotypes in the modern sheep breeds by sequencing samples of 10 Eurasian sheep breeds and by searching for shared sequences in the GenBank DNA database. The shared haplotype analysis showed that 14 of the 18 ancient haplotypes were present in the modern sheep breeds and in the GenBank DNA database (Figure 1) and [Additional file 3: Table S2]. In the modern sheep populations, frequencies of ancient haplotypes were highest in the native Finnish sheep breeds, Finnsheep (0.38) and Kainuu Grey (0.53) with six ancient haplotypes i.e. H11 in haplogroup A and H22, H32, H40, H42, H44 in haplogroup B. The two ancient haplotypes, H05 in haplogroup A and H32 in haplogroup B are common haplotypes while other rarer ancient haplotypes exist e.g. in contemporary native breeds from the Caucasus, Russia and Serbia [see Additional file 3: Table S2]. The four ancient haplotypes H06, H30, H43 and H50 were absent both in our own modern dataset and in the GenBank DNA database [see Additional file 3: Table S2].

### Analysis of population diversity

The statistics summarising the level of mtDNA variation in the Finnish ancient populations and modern sheep breeds and in seven other modern Eurasian breeds are presented in Tables 2 and 3, respectively. Additional file 1: Figure S1 shows the sites from which the Finnish archaeological samples were collected and for which mtDNA was successfully amplified. The Iron Age cohort comprising only five samples and excavated at a single archaeological site on the prehistorical farm Mulli displayed the lowest values for all diversity estimates (s, h, Hd, K, and π). Haplotype diversity was higher in ancient sheep samples from the Medieval and Post-Medieval periods (Hd = 1.0 and 0.98, respectively) than in any of the modern populations. The Post-Medieval samples originated from several archaeological sites, while the Medieval samples were excavated mainly in Turku. Among the modern sheep breeds analysed, Bozakh, Finnsheep and Pramenka showed the highest haplotype diversity (Hd > 0.90; Tables 2 and 3) while Olkuska and Åland had the lowest haplotype diversity (Hd = 0.75; Tables 2 and 3). The Finnsheep samples were collected in several sheep flocks from different parts of Finland. The measures taking in account the molecular nature of the data showed highest variation in the populations and breeds in which both haplogroups A and B were segregating. Nucleotide diversity varied among cohorts and was highest (π = 15.51 * 10^-3^) in Pramenka sheep, originating from the Balkan region, which was one of the main dispersal routes for Near Eastern domesticated sheep entering Europe.

The diversity estimate θs is influenced by genetic bottlenecks, whereas θπ is relatively insensitive. Consequently, θπ-θs will be negative in stable populations under an infinite-sites model of mutation-drift equilibrium and positive as a result of a reduction in the number of segregating sites. In our data, θπ-θs estimates were negative i.e. -1.465 and −1.563 for Post Medieval and modern Finnsheep, respectively (Table 2), whereas they were positive for Åland, Kainuu Grey, Olkuska, and Pramenka sheep, and slightly positive for the Pramenka breed and the Medieval population (Tables 2 and 3). Statistically significant Tajima’s D-values were positive for Kainuu Grey and Olkuska sheep, indicating a reduction of mtDNA diversity.

### Y-chromosome analysis

**Figure 2:**
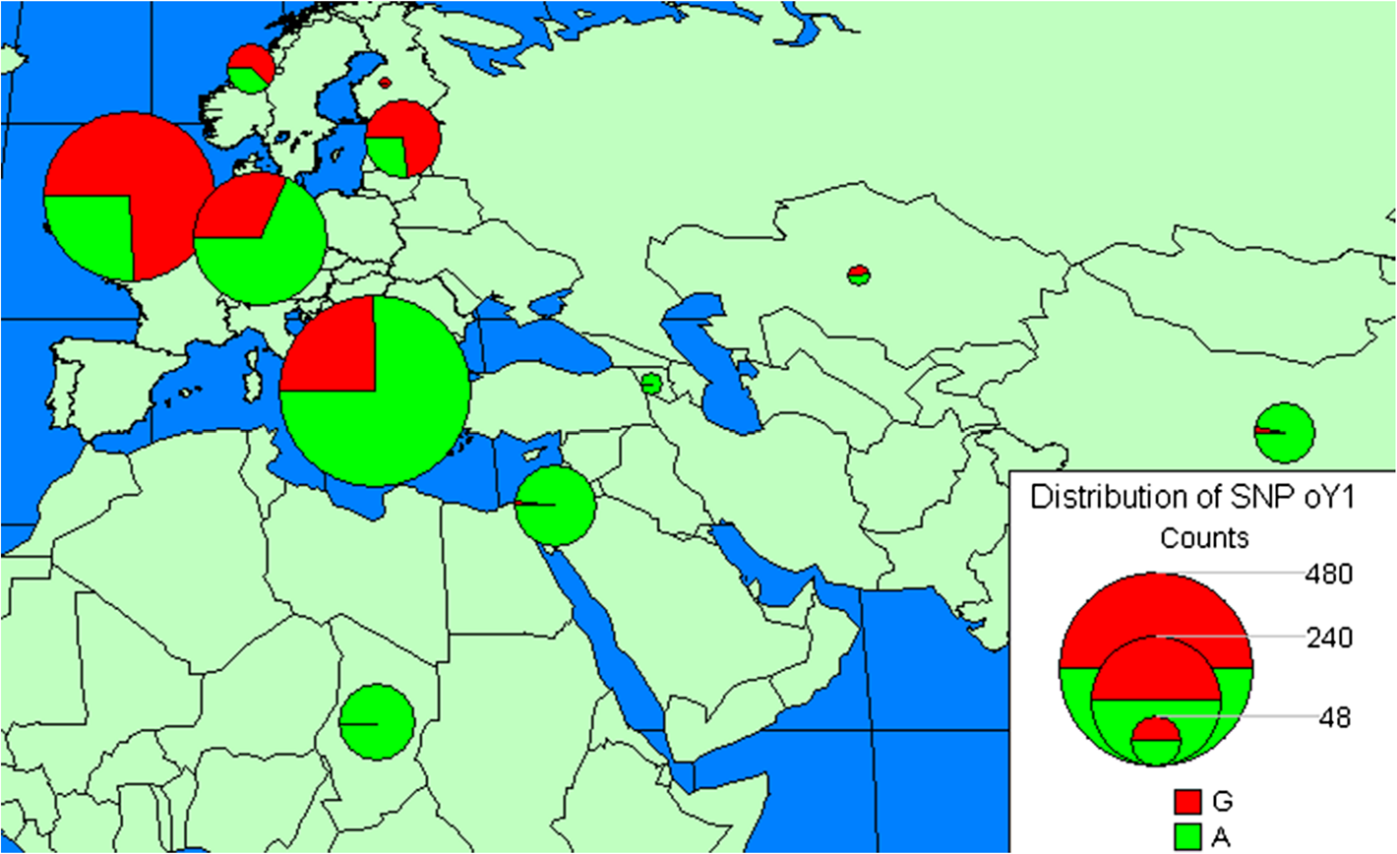
**Distribution of Y-chromosomal SNP G*- *and *A-oY *1 in modern sheep breeds in Europe, Asia and Africa and in ancient Finnish sheep.** Ancient sheep are indicated by a smaller circle over Finland at the northern most location on the map; the remaining circles on the map indicate modern sheep breeds from [9,12] listed in [Additional file 4: Table S3]; the proportions of G-*oY*1 and A-*oY*1 haplotypes are indicated in red and green, respectively; the size of the circles is proportional to sample size in each location.

For three of the 27 ancient sheep samples, Y-chromosome *SRY* sequences were amplified and were thus genetically identified as rams (Table 1) with one ram sample from the Medieval period and two from the Post-Medieval period (Table 1). All three ancient Finnish sheep had SNP G-*oY*1 in the *SRY* gene. In the modern Finnish breeds, the frequency of G-*oY*1 was 77%, 57%, and 60% in Finnsheep, Kainuu Grey, and Åland, respectively [see Additional file 4: Table S3], indicating that SNP G-*oY1* is frequent in the modern north-European breeds (Figure 2).

## Discussion

We successfully analysed mtDNA and Y-chromosome diversity in ancient sheep remains from Finland and found that Finnish sheep genetic diversity has been quite constant over the last 1000 years. Our aDNA results fit well with the genetic context of the modern north-European domestic sheep breeds analysed either previously [3,12] or in the present study. Both ovine haplogroups A and B have been present in the Finnish sheep population for at least more than 700 years and no remarkable temporal changes in their frequencies have occurred. Four of the 26 ancient mtDNA sequences obtained here were assigned to haplogroup A (the frequency in different ancient cohorts varied from 0 to 28.6%) and 22 sequences to haplogroup B (frequency ranging between 71.4 and 100%). The respective haplogroup A and B frequencies in the present sample set of three Finnish native breeds are 21.9% and 78.1% according to our analysis and 16.7% and 83.3% according to [3]. Four of the 18 ancient mtDNA haplotypes were present in the modern Finnish native breeds and 14 in the set of 10 Eurasian breeds analysed here. The affinity between modern mtDNA haplotypes and the ancient mtDNA haplotypes not found in the modern breeds is also attested by their proximity within the phylogeny and haplotype network in Figure 1 and Figure S3 [see Additional file 6: Figure S3], respectively. According to historical written records, foreign breeding animals, which were obviously ancestors of the modern Merino sheep, were imported into Finland during the 16th, 17th and 18th centuries [8,56]. However, most of the imported individuals being rams, they did not have a major impact on the mtDNA diversity in the Finnish native sheep.

In addition, results of the Y-chromosome analysis on aDNA agree well with those on DNA from modern Finnish indigenous sheep breeds. SNP G-*oY*1 in the 5’-promoter region of the *SRY* gene on sheep Y-chromosome was detected in the three ancient ram samples. Since these ancient ram samples (OaNaa1, OaTor1, OaKök2, Table 1) were collected from different excavation sites and from two different time periods (Medieval and Post-Medieval), it is unlikely that they were close relatives. However, the number of available ancient ram samples is not sufficient to draw detailed conclusions on the temporal changes in the frequencies of G-*oY*1 and A-*oY1*. In the contemporary native Finnish breeds, the frequency of SNP G-*oY*1 ranges from 57 to 77% [see Additional file 4: Table S3] and the frequency of SNP G-*oY*1 is highest in north European, British Islands and central Russian populations [12], [see Figure 2 and Additional file 4: Table S3]. SNP G-*oY*1 is less frequent in southern and central European sheep (25% and 32%, respectively) and very low for the remaining breeds analysed to date (< 8%) [see Figure 2 and Additional file 4: Table S3]. The presence of SNP G-*oY*1 in modern sheep populations often reflects introgression of English breeds [12]. However, this cannot be the case for ancient Finnish sheep. Consequently, our finding that SNP G-*oY*1 is present in sheep aDNA suggests that (at least in Finland) this paternal line predates the arrival of sheep in northern Europe.

Our results on radiocarbon-dating of 19 sheep remains excavated from archaeologically important sites in Finland provide essential information for Finnish archaeological research. Our research on ancient sheep material shows that previous attempts to determine the age of remains based on archeological context are fairly accurate, since only in three cases, did the dating results differ from those expected (OaPih1, OaKök2 and OaUuk2, Table 1). The oldest samples analysed here originate from the Late Iron Age from the excavation site Mulli in Raisio and from Pasvik in northern Norway. In Finnish archaeology, age determination of specimens is usually based on archaeological context. However, this can be misleading because, in Finland, cultural layers are thin and younger bone and other animal materials may have sunk to earlier cultural layers and thus, the dating of a single bone might differ from the general dating of a site [20]. Therefore, the dating of archaeological materials by radiocarbon methods is recommended.

Here, we investigated the genetic affinities between the ancient Finnish sheep populations and the modern sheep breeds by searching for identical matches in the GenBank DNA database with the ancient haplotype sequences. Comparison of our ancient mtDNA data with those of the contemporary breeds appears to be relatively uninformative in terms of unfolding geographic origin or origins of native sheep in Finland or the dispersal of sheep husbandry to Finland. Our results confirm that the domestic sheep populations share their origins with *O. orientalis* populations domesticated in the Near Eastern region, from where the sheep spread around the World, and that whereas haplogroup B is common in Europe, haplogroup A is much rarer [1-3]. Ancient mtDNA haplotypes can be found in modern sheep breeds originating from geographically distant regions. Ancient Finnish sheep show a maternal genetic affinity to western and southern European breeds, but also to eastern European breeds. For example, haplotype H05 detected in the Post-Medieval sheep is also present in the two Russian (Figure 1) and the Iberian sheep breeds [see Additional file 3: Table S2] and several haplotypes of the haplogroup B detected in the Finnish ancient sheep are present in eastern, western and southern European breeds. However, four mtDNA haplotypes present in the ancient sheep samples are absent in the contemporary sheep breeds suggesting a possible loss of these haplotypes. Our results agree with a previous analysis of mtDNA D-loop polymorphisms on modern Eurasian sheep breeds in which a genetic historical influence of Russian sheep breeds in northern European sheep breeds was detected [3].

We assume that the fluctuations in mtDNA genetic diversity estimates obtained for the different cohorts (Tables 2 and 3) may be stochastic in nature as a result of genetic drift and sampling. Assuming neutrality for the mtDNA D-loop region, the high positive values for (θπ-θs) indicate a loss of genetic variability in terms of number of segregating nucleotide sites, occurring in the modern populations of Åland, Kainuu Grey, Olkuska sheep and the Serbian Pramenka population of Vlashko Vitoroga. These breeds experienced a genetic bottleneck during the 20th century and are classified as endangered sheep breeds [14]. In addition, their positive Tajima’s D-values – statistically significantly different from zero for Kainuu Grey and Olkuska sheep – point towards an effect of a decline of population size on mtDNA diversity. In contrast, our within-population diversity estimates for Finnsheep, which is a large, major sheep population in Finland and having descended during the 20th century from a broad founder population, do not indicate loss of mtDNA diversity in haplogroup B. However, our Finnsheep data can be considered slightly biased because we could not detect haplogroup A in our sample set [3]. When the diversity estimates of the ancient samples are compared with those of the modern breeds, values for (θπ-θs) and Tajima’s D in the Medieval sheep population are similar to estimates that could be obtained for an endangered modern breed having experienced a reduction in population size. In contrast, the estimates for the Post-Medieval cohort are similar to those of a modern large sheep breed with a growing population size. However, the origin and availability of archaeological materials could explain these differences in estimates rather than demographic events that occurred in the past. The Medieval samples are mainly from one region, the Turku region, while Post-Medieval samples are from a wider geographic region, from southern, northern, western and central parts of Finland. The present Iron Age samples displaying a lower level of mtDNA diversity are from the Mulli farm from Raisio and four of the samples share the same maternal ancestry. Interestingly, it appears that the same sheep ‘dam-lineage’ was raised in Mulli for a long period and no new ewes were introduced into the flock.

This aDNA study focusing on sheep biodiversity in Finland shows that population genetic analysis of ancient domestic animal populations and studies investigating changes in genetic diversity across different eras are challenging. The availability of ancient materials and the success rate of ancient DNA analysis have a decisive impact on how comprehensively a sample set represents the genetic variation of ancient animal populations. In the Finnish context, survival of unburned bones from time periods prior to the Late Iron Age is infrequent due to the acid centeracter of soils. In addition, in our study, the Medieval and Post-Medieval samples are not from the same temporal population as the samples of the modern breeds, but mainly from temporally distant generations. For example, the age difference between the oldest and youngest Medieval samples is approximately 300 years, corresponding to a time span of 100 generations. The population structures of ancient and modern cohorts are different, interfering with conclusions on temporal changes between ancient and modern populations. Moreover, the 300 years long Medieval period or Iron Age period were not socially or culturally static and our research area can be roughly divided into western, eastern and northern cultural regions with their own cultural and trade networks possibly influencing the genetic variation and structure of ancient animal populations. More ancient materials are needed to examine the archaeological questions in more detail.

## Conclusions

To date, mitochondrial DNA has been the most popular genetic marker to centeracterize ancient farm animal populations, to trace their ancestor and reconstitute the number of domestication events. Less is known about Y-chromosome polymorphisms in ancient domestic animals partly due to challenges in Y-chromosome marker typing and partly due to the lack of polymorphic markers currently available for centeracterization. Here we have published one of the most comprehensive mtDNA analysis in ancient sheep populations to date and simultaneously successfully typed the Y-chromosome SNP marker *oY*1 in three ancient sheep samples. Our aDNA results are in agreement with the previous international studies on mtDNA and Y-chromosome diversity of domestic sheep. However, for more detailed archaeological and archaeozoological studies, such as regional historical dispersal of animal populations, and origins and relatedness of ancient animals found in a same excavation site or different sites, the resolving power of mtDNA can be too low. Our mtDNA study indicates that the same ancient and modern ovine mtDNA haplotypes can be detected in geographically distant regions. As indicated by the analysis of modern sheep breeds e.g. [9,14], autosomal nuclear markers, such as microsatellites, sequence data and SNP are powerful population genetic markers. Further studies with these autosomal nuclear markers will offer an opportunity to study archaeological samples in more detail and provide more information about ancient genetic population structure than uniparentally inherited markers.

## Competing interests

The authors declare that they have no competing interests.

## Authors’ contributions

MN carried out the molecular genetic studies of ancient and modern DNA, MN and JK performed the statistical analysis and drafted the manuscript. AB and JH selected the ancient samples for the study and drafted the archaeological and historical parts of the manuscript. VN and TI-T participated in molecular genetic studies and the design of the laboratory methods. KL provided the education for aDNA analyses at one of the two aDNA laboratories used in the study. TI-T took part also in the statistical analyses and the manuscript writing. JK, J-PT and JS conceived the study, and participated in its design and coordination and helped to draft the manuscript. All authors read and approved the final manuscript.

## Supplementary Material

Additional file 1 ** Figure S1.** Title: Sample sites for ancient and modern sheep. Description: This figure presents excavation sites of ancient sheep samples included in statistical analysis. Click here for file

Additional file 2 **Table S1.** Title: Primers used in this study. Description: This table presents primer pairs, annealing temperatures (AT), fragment length, nucleotide position of initiation of the amplification and average amplification success rates for aDNA and modern samples. Click here for file

Additional file 3 **Table S2.** Title: Distribution of haplotypes identified in the present study and in data available from GenBank. Description: The table provides distribution of haplotypes found in this study among previously studied contemporary sheep populations [2,57-63] available in GenBank. Click here for file

Additional file 4 **Table S3.** Title: Global distribution of Y chromosomal SNPs A- *oY1* and G-*oY1*. Description: The table provides distribution of *oY1* SNPs in modern sheep breeds according to [9,12]. Three ancient Finnish sheep analysed in this study are added to the data. Click here for file

Additional file 5 **Figure S2.** Title: Mitochondrial DNA haplotypes (H) identified in 26 ancient and 94 modern sheep. Description: The data provided represent alignment of 26 ancient and 94 modern sheep analysed in this study. SNP positions given are relative to the reference sequence [GenBank:NC001941]. Click here for file

Additional file 6 **Figure S3 and S4.** Title: Median-joining network and mismatch distribution of the 56 mitochondrial haplotypes. Description: Figure S3 shows the median-joining network (ε = 0) with molecular relationships between 56 haplotypes which cluster into two major ovine haplogroups: haplogroup A (H01-H14, on the left) and haplogroup B (H15-H56, on the right). Figure S4 presents the mismatch distribution of 120 modern and ancient domestic sheep indicating the existence of two divergent haplogroups in the data. Click here for file
