# Supplementary material for: Mitochondrial DNA and Y-chromosomal diversity in ancient populations of domestic sheep (Ovis aries) in Finland: comparison with contemporary sheep breeds
Source: Genet Sel Evol. 2013 Jan 22;45(1):2. doi: 10.1186/1297-9686-45-2 (PMC3558444; doi:10.1186/1297-9686-45-2)
Supplement: Additional file 1 — Figure S1. Title: Sample sites for ancient and modern sheep. Description: This figure presents excavation sites of ancient sheep samples included in statistical analysis. [file 1297-9686-45-2-S1.docx]

**Additional file 1, Figure S1**

Excavation sites for Ancient sheep samples in Finland and one site in Northern Norway from Iron Age (in black) Medieval (in orange) and Post-Medieval (in green) periods and origin of modern sheep breeds (in blue) are indicated; only the excavation sites of the samples included in the statistical analyses are shown (for more information see the text). Modern sheep breeds and country of origin are: Oxford down from England (sampled in Finnish pure breed subpopulation); Olkuska from Poland; Pramenka from Serbia; Bozakh from Caucasus; Oparino and Romanov from central Russia; Viena sheep from north-westen Russia; Kainuu Grey Sheep and Finnsheep from mainland Finland; Åland sheep from island of Åland located South-West from Finland.
