# Supplementary material for: Mitochondrial DNA and Y-chromosomal diversity in ancient populations of domestic sheep (Ovis aries) in Finland: comparison with contemporary sheep breeds
Source: Genet Sel Evol. 2013 Jan 22;45(1):2. doi: 10.1186/1297-9686-45-2 (PMC3558444; doi:10.1186/1297-9686-45-2)
Supplement: Additional file 2 — Table S1. Title: Primers used in this study. Description: This table presents primer pairs, annealing temperatures (AT), fragment length, nucleotide position of initiation of the amplification and average amplification success rates for aDNA and modern samples. [file 1297-9686-45-2-S2.doc]

### Additional file 2, Table S1

### Primers used in this study, annealing temperatures (AT), fragment length, nucleotide position of initiation of the amplification and average amplification success rates for aDNA and modern samples. Nucleotide positions showing the mtDNA and *SRY* gene amplifications are given according to the reference sequences NC_001941 and AF026566, respectively. Y-chromosomal SRY gene primers were described in [9].

| Fragment | Primer pair | AT | Fragment length | Start Position (according to NC_001941) | **Average amplification success** |
| --- | --- | --- | --- | --- | --- |
| D-loop fragment 1 | For 5’GTTTCACTGAAGCATGTAGGG3’  Rev 5’CATGGTGAACAAGCTCGTGA3’ | 58 | 116 | 15957 | 0.68 |
| D-loop fragment 2 | For 5’TCAACATGCGTATCCTGTCC3’  Rev 5’ATGGCCCTGAAGAAAGAACC3’ | 60 | 164 | 16027 | 0.65 |
| D-loop fragment 3 | For 5’CCCATTAACTGTGGGGGTAA3’  Rev 5’AATACCAAATGCATGACACCA3’ | 58 | 172 | 16124 | 0.66 |
| D-loop fragment 4 | For 5’TCAGCCCATGCCTAACATAA3’  Rev 5’TGAGGATGCTCAAGATGCAG3’ | 58 | 143 | 16252 | 0.47 |
| D-loop fragment 5 | For 5’CGGAGCATGAATTGTAGCTG3’  Rev 5’GTATTGAGGGCGGGATAAAT3’ | 58 | 208 | 16348 | 0.45 |
| *Ovies aries* SRY | For 5’AGCTCCAGAATATTTCACTGACCT3’  Rev 5’GAAGGCAAATGCAGAGACAA3’ | 58 | 130 | 3103 (according to AF026566) | 0.12 |
| D-loop for modern samples | For 5’GTTTCACTGAAGCATGTAGGG3’  Rev 5’GTATTGAGGGCGGGATAAAT3’ | 58 | 523 | 15957 |  |
