# Supplementary material for: Mitochondrial DNA and Y-chromosomal diversity in ancient populations of domestic sheep (Ovis aries) in Finland: comparison with contemporary sheep breeds
Source: Genet Sel Evol. 2013 Jan 22;45(1):2. doi: 10.1186/1297-9686-45-2 (PMC3558444; doi:10.1186/1297-9686-45-2)
Supplement: Additional file 3 — Table S2. Title: Distribution of haplotypes identified in the present study and in data available from GenBank. Description: The table provides distribution of haplotypes found in this study among previously studied contemporary sheep populations [2,57-63] available in GenBank. [file 1297-9686-45-2-S3.doc]

**Additional file 3, Table S2**

Distribution of haplotypes identified in the present study and in data available from GenBank. Only the haplotypes shared between our data and the data from one or more breeds available in GenBank are shown. Analyses of GenBank data can be found in the references indicated in the column on the right.

| **breed and location** | **Origin** | **H**  **05** | **H**  **06** | **H**  **09** | **H**  **11** | **H**  **15** | **H**  **22** | **H**  **24** | **H**  **25** | **H**  **26** | **H27** | **H**  **31** | **H**  **32** | **H**  **33** | **H**  **36** | **H**  **38** | **H**  **39** | **H**  **40** | **H**  **41** | **H**  **42** | **H**  **43** | **H44** | **H**  **46** | **H**  **48** | **H**  **51** | **H**  **52** | **H**  **55** | **Reference** |
| --- | --- | --- | --- | --- | --- | --- | --- | --- | --- | --- | --- | --- | --- | --- | --- | --- | --- | --- | --- | --- | --- | --- | --- | --- | --- | --- | --- | --- |
| Tibetan domestic sheep breed | China | 2 |  |  |  |  |  |  |  |  |  |  |  |  |  |  |  |  |  |  |  |  |  |  |  |  |  | [38] |
| Ganjia sheep, Gansu province, China | China | 1 | 1 |  |  |  |  |  |  |  |  |  |  |  |  |  |  |  |  |  |  |  |  |  |  |  |  | [39] |
| Hu, Chinese local breed | China |  |  |  |  |  |  |  |  |  |  |  | 1 |  |  |  |  |  |  |  |  |  |  |  |  |  |  | [40] |
| Mongolian, Chinese local breed | China | 2 |  |  |  |  |  |  |  |  |  |  |  |  |  |  |  |  |  |  |  |  |  |  |  |  |  | [40] |
| Tibetan domestic sheep breed | China | 2 |  |  |  |  |  |  |  |  |  |  |  |  |  |  |  |  |  |  |  |  |  |  |  |  |  | [38] |
| Lanzhou big tail sheep, Gansu province | China | 1 |  |  |  |  |  |  |  |  |  |  |  |  |  |  |  |  |  |  |  |  |  |  |  |  |  | [39] |
| Oula sheep, Gansu province | China | 2 |  |  |  |  |  |  |  |  |  |  |  |  |  |  |  |  |  |  |  |  |  |  |  |  |  | [39] |
| Small Tailed Han, Chinese local breed | China | 2 |  |  |  |  |  |  |  |  |  |  |  |  |  |  |  |  |  |  |  |  |  |  |  |  |  | [40] |
| Chinese breed | China | 2 |  |  |  |  |  |  |  |  |  |  |  |  |  |  |  |  |  |  |  |  |  |  |  |  |  | [39] |
| Tashikuergan sheep, Xinjiang province | China | 1 |  |  |  |  |  |  |  |  |  |  |  |  |  |  |  |  |  |  |  |  |  |  |  |  |  | [39] |
| Astrachan, Kazakhstan, Tschimkent | Central Asia | 1 |  |  |  |  |  |  |  |  |  |  |  |  |  |  |  |  |  |  |  |  |  |  |  |  |  | [41] |
| Gizarr, Tadjikistan | Central Asia | 1 |  |  |  |  |  |  |  |  |  |  |  |  |  |  |  |  |  |  |  |  |  |  |  |  |  | [41] |
| Oparino, Russia | Central Asia | 2 |  |  |  |  |  |  |  |  |  | 1 | 3 | 1 |  |  |  |  |  |  |  |  |  |  |  | 2 |  | This study |
| Romanov, Russia | Central Asia |  |  |  |  |  |  |  |  |  |  | 2 | 2 |  |  |  |  |  |  |  |  |  |  |  |  |  |  | This study |
| Bozakh, Caucasus | Caucasus | 2 |  |  |  |  |  |  |  |  |  |  |  |  |  |  |  |  |  |  |  |  |  |  |  |  |  | This study |
| Spanish Assaf mix | Near East |  |  |  |  |  |  |  |  |  |  |  | 1 |  |  |  |  |  |  |  |  |  |  |  |  |  |  | [42] |
| Awassi and Improved Awassi, Israel | Near East | 2 |  |  |  |  |  |  |  |  |  |  |  |  |  |  |  | 1 |  |  |  |  |  |  |  | 1 |  | [42, 43] |
| Ossimi, Egypt | Near East | 1 |  |  |  |  |  |  | 1 |  |  |  |  |  | 1 |  |  |  |  |  |  |  |  |  |  |  |  | [42] |
| Rahmani, Egypt | Near East |  |  |  |  |  |  |  |  |  |  |  | 1 |  |  |  |  |  |  |  | 1 |  |  |  | 1 |  |  | [42] |
| Akkaraman, Turkey | Turkey |  |  |  |  |  |  |  |  |  |  |  | 2 |  |  |  |  |  |  |  |  |  |  |  |  |  |  | [44] |
| Hemshin, Turkey | Turkey | 1 |  |  |  |  |  |  |  |  |  |  |  |  |  |  |  |  |  |  |  |  |  |  |  |  |  | [44] |
| Karayaka, Turkey | Turkey |  |  | 1 |  |  |  |  |  |  |  |  | 1 |  |  |  |  |  |  |  |  |  |  |  |  |  |  | [44] |
| Karakas, province of Van, eastern Turkey | Turkey |  |  |  |  |  |  |  |  |  |  |  | 2 |  |  |  |  |  |  |  |  |  |  |  |  |  |  | [43] |
| Morkaraman, Turkey | Turkey |  |  |  |  |  |  |  |  |  |  |  |  |  |  |  |  |  |  |  |  |  |  |  |  |  |  | [44] |
| Turkey | Turkey | 6 |  | 1 |  |  |  |  |  |  |  | 1 | 3 |  |  |  |  |  | 1 |  |  |  |  |  |  |  |  | Koban et al. Unbublished |
| Tuj, Turkey | Turkey |  |  |  |  |  |  |  |  |  |  |  | 1 |  |  |  |  |  |  |  |  |  |  |  |  |  |  | [44] |
| Lacaune, France | Central Europe |  |  |  |  |  |  |  |  |  |  |  | 2 |  |  |  |  |  |  |  |  |  |  |  |  |  |  | [42] |
| Milchschaf, Germany | Central Europe |  |  |  |  |  | 1 |  |  |  |  |  |  |  |  |  |  |  |  |  |  |  |  |  |  |  |  | [42] |
| Olkuska, Poland | Central Europe |  |  |  |  |  |  |  | 1 |  |  |  | 1 |  |  |  |  |  |  |  |  |  |  |  |  |  |  | This study |
| Oxford Down, UK, Finnish subpopulation | Central Europe |  |  |  |  |  |  | 1 |  |  |  |  |  |  |  | 2 |  |  |  |  |  |  |  |  |  |  |  | This study |
| Polled Dorset, Western breed, Chinase subpopulation | China/ Western breed | 1 |  |  |  |  |  |  |  |  |  |  | 2 |  |  |  |  |  |  |  |  |  |  |  |  |  |  | [40] |
| Texel, Western breed, Chinase subpopulation | China/ Western breed |  |  |  | 1 |  |  |  |  |  |  |  |  |  | 1 |  |  |  |  |  |  |  |  |  |  |  |  | [40] |
| Pramenka, Serbia | Central Europe |  |  | 2 |  |  |  |  |  |  | 1 |  |  |  |  |  |  | 1 |  |  |  |  |  |  |  |  | 1 | This study |
| **Table S2**  **Continued**  **breed and location** | **Origin** | **H**  **05** | **H**  **06** | **H**  **09** | **H**  **11** | **H**  **15** | **H**  **22** | **H**  **24** | **H**  **25** | **H**  **26** | **H27** | **H**  **31** | **H**  **32** | **H**  **33** | **H**  **36** | **H**  **38** | **H**  **39** | **H**  **40** | **H**  **41** | **H**  **42** | **H**  **43** | **H44** | **H**  **46** | **H**  **48** | **H**  **51** | **H**  **52** | **H**  **55** | **Reference** |
| Åland Sheep, Finland | North Europe |  |  |  |  |  |  | 4 |  |  |  |  |  |  |  | 1 |  |  |  |  |  |  |  |  |  |  |  | This study |
| Kainuu Grey Sheep, Finland | North Europe |  |  |  | 3 | 2 |  |  |  |  |  |  |  |  |  |  |  |  | 2 |  |  |  |  |  |  |  |  | This study |
| Finnsheep, Finland | North Europe |  |  |  |  |  | 1 |  |  |  |  |  | 3 | 3 |  |  |  | 2 |  | 1 |  | 1 | 1 |  |  |  |  | This study |
| Ancient sheep from Iron Age, Finland | North Europe |  |  |  |  |  |  |  |  |  |  |  | 1 |  |  |  |  |  |  |  |  |  |  |  |  |  |  | This study |
| Ancient sheep from Medieval, Finland | North Europe |  | 1 |  | 1 |  | 1 |  |  | 1 | 1 |  |  |  |  |  |  |  |  | 1 |  |  |  | 1 |  |  |  | This study |
| Ancient sheep from Post Medieval, Finland | North Europe | 1 |  |  |  |  | 1 |  |  |  | 1 |  | 1 |  | 1 |  |  | 2 |  |  | 2 | 1 |  |  | 1 |  |  | This study |
| Viena, West Russia | North Europe |  |  |  |  | 1 |  |  |  |  |  |  | 1 |  |  |  | 1 |  |  |  |  |  | 2 |  |  |  |  | This study |
| Alcarreña, Iberia, ENTREFINO TRUNK | Iberia |  |  |  |  |  |  |  |  |  |  |  | 2 |  |  |  |  |  |  |  |  |  |  |  |  |  |  | [42] |
| Churra Algarvia, Churra type, Portugal | Iberia |  |  |  |  |  | 1 |  | 2 |  |  |  | 8 |  |  |  |  |  |  |  |  |  |  |  |  |  | 1 | [45] |
| Rasa Aragonesa, Iberia, ENTREFINO TRUNK | Iberia |  |  |  |  |  |  |  |  |  |  |  |  |  |  |  |  |  |  |  |  |  |  | 1 |  |  |  | [42] |
| Churra Badana, Churra type, Portugal | Iberia | 1 |  |  |  |  |  |  | 2 |  |  |  | 2 |  |  |  |  |  |  |  | 1 |  |  |  |  |  |  | [45] |
| Chu. Ga. Bragançana, Iberia, CHURRO TRUNK | Iberia |  |  |  |  |  |  |  |  |  |  |  | 1 |  |  |  |  |  |  |  |  |  |  |  |  | 1 |  | [42] |
| Campaniça, Bordaleiro type, Portugal | Iberia |  |  |  |  |  |  |  |  |  |  | 1 | 3 |  |  |  |  | 2 |  | 1 | 1 |  |  |  |  |  |  | [45] |
| Castellana, Iberia, ENTREFINO TRUNK | Iberia |  |  |  |  |  |  |  | 2 |  |  |  | 1 |  |  | 2 |  | 1 |  |  |  |  |  |  |  |  |  | [42] |
| Churra, Iberia, CHURRO TRUNK | Iberia |  |  |  |  |  |  |  |  |  |  |  |  |  |  |  |  | 1 |  | 1 |  |  |  | 1 |  |  |  | [42] |
| Latxa, Iberia, CHURRO TRUNK | Iberia |  |  |  |  | 1 |  |  |  |  |  |  |  |  |  |  | 1 |  |  |  |  |  |  |  |  |  |  | [42] |
| Manchega, Iberia, ENTREFINO TRUNK | Iberia | 1 |  |  |  |  |  |  |  |  |  |  | 1 |  |  |  |  |  |  |  |  |  |  |  | 1 |  |  | [42] |
| Merino de Beira Baixa, Iberian, MERINO TRUNK | Iberia |  |  |  |  |  |  |  |  | 1 |  |  |  | 1 |  |  |  |  |  |  |  |  |  |  |  |  |  | [42] |
| Merino Branco, Iberian, MERINO TRUNK | Iberia |  |  |  |  | 1 |  |  |  |  |  |  |  |  |  |  |  |  |  |  |  |  |  |  |  |  |  | [42] |
| Mondegueira, Churra type, Portugal | Iberia | 1 |  |  |  |  |  |  |  |  |  |  | 1 |  |  |  |  |  |  |  |  |  |  |  |  |  |  | [45] |
| Spanish Merino, Iberian, MERINO TRUNK | Iberia |  |  |  |  |  |  |  |  |  |  |  | 1 |  |  |  |  |  |  |  |  |  |  |  |  |  |  | [42] |
| Montesina, Iberia, IBERIAN TRUNK | Iberia |  |  |  |  |  |  |  |  |  |  |  | 1 |  |  |  |  |  |  |  |  |  |  |  |  |  |  | [42] |
| Ojalada, Iberia, IBERIAN TRUNK | Iberia |  |  |  |  |  |  |  |  |  |  |  |  |  |  |  |  |  |  |  |  |  |  |  |  |  |  | [42] |
| Merino Preto, Merino type, MERINO TRUNK,Portugal | Iberia |  |  |  |  |  |  |  |  |  |  |  | 1 |  |  |  |  |  |  |  | 1 |  |  |  |  |  |  | [42, 45] |
| Saloia, Bordaleiro type, Portugal | Iberia |  |  |  |  |  | 1 |  |  |  |  |  | 4 |  |  |  |  |  |  |  | 2 |  |  |  |  |  |  | [45] |
| Serra da Estrela, Iberia, ENTREFINO TRUNK | Iberia |  |  |  |  |  |  |  |  |  |  |  | 1 |  |  |  |  |  |  |  |  |  |  |  |  |  |  | [42] |
| Segureña, Iberia, ENTREFINO TRUNK | Iberia |  |  |  |  |  |  |  |  |  |  |  |  |  |  |  | 1 |  |  |  |  |  |  |  |  |  |  | [42] |
| Churra da Terra Quente, Churra type, Portugal | Iberia |  |  |  |  |  |  |  |  | 1 |  |  | 3 |  |  |  |  |  |  |  |  |  |  |  |  | 1 |  | [45] |
| Xalda, Iberia, CHURRO TRUNK | Iberia |  |  |  |  |  |  |  |  |  |  |  |  |  |  |  |  |  |  |  |  |  |  |  |  |  |  | [42] |
| Merino de Grazalema | Iberia |  |  |  |  |  |  |  |  | 1 |  |  | 1 |  |  |  |  |  |  |  |  |  |  |  |  |  |  | [42] |
