# Supplementary material for: Mitochondrial DNA and Y-chromosomal diversity in ancient populations of domestic sheep (Ovis aries) in Finland: comparison with contemporary sheep breeds
Source: Genet Sel Evol. 2013 Jan 22;45(1):2. doi: 10.1186/1297-9686-45-2 (PMC3558444; doi:10.1186/1297-9686-45-2)
Supplement: Additional file 4 — Table S3. Title: Global distribution of Y chromosomal SNPs A- oY1 and G-oY1. Description: The table provides distribution of oY1 SNPs in modern sheep breeds according to [9,12]. Three ancient Finnish sheep analysed in this study are added to the data. [file 1297-9686-45-2-S4.doc]

Additional file 4, Table S3

Global distribution of Y chromosomal SNPs A- *oY*1 and G-*oY*1.

The distribution of *oY*1 SNPs in modern sheep breeds is according to [9,12]. Three ancient Finnish sheep analysed in this study are added to the data.

|  |  |  | SNP ***oY1*** |  |  |  |  |
| --- | --- | --- | --- | --- | --- | --- | --- |
| **Region** | **Breeds** | **(n)** | ***oY*1-G** |  | ***oY1*-A** |  | **Reference** |
| Africa |  |  |  |  |  |  |  |
|  | African Doper | 2 | 0 | 0 % | 2 | 100 % | [9] |
|  | Ethiopian Menz | 18 | 0 | 0 % | 18 | 100 % | [9] |
|  | Namaqua Afrikaner | 8 | 0 | 0 % | 8 | 100 % | [9] |
|  | Red Maasai | 11 | 0 | 0 % | 11 | 100 % | [9] |
|  | Ronderib Afrikaner | 17 | 0 | 0 % | 17 | 100 % | [9] |
|  | Arsi Bale | 5 | 0 | 0 % | 5 | 100 % | [12] |
|  | Balami | 9 | 0 | 0 % | 9 | 100 % | [12] |
|  | Pedi | 5 | 0 | 0 % | 5 | 100 % | [12] |
|  | Sabi | 5 | 0 | 0 % | 5 | 100 % | [12] |
|  | Sukuma | 5 | 0 | 0 % | 5 | 100 % | [12] |
|  | Tswana | 4 | 0 | 0 % | 4 | 100 % | [12] |
|  | West African Dwarf Djallo | 9 | 0 | 0 % | 9 | 100 % | [12] |
|  | **Africa Total** | **98** | **0** | **0** % | **98** | **100** % |  |
| Eastern Asia | |  |  |  |  |  |  |
|  | Tibetan | 41 | 2 | 5 % | 39 | 95 % | [9,12] |
|  | Javanese Thin Tail | 5 | 0 | 0 % | 5 | 100 % | [12] |
|  | Mongolian | 3 | 0 | 0 % | 3 | 100 % | [12] |
|  | **Eastern Asia Total** | **49** | **2** | **4** % | **47** | **96** % |  |
| North-West Asia | |  |  |  |  |  |  |
|  | Edilbaevskaya | 2 | 0 | 0 % | 2 | 100 % | [12] |
|  | Karakul | 4 | 0 | 0 % | 4 | 100 % | [12] |
|  | Kuibyshev | 5 | 5 | 100 % | 0 | 0 % | [12] |
|  | **North-West Asia total** | **11** | **5** | **45 %** | **6** | **55 %** |  |
| South Asia | |  |  |  |  |  |  |
|  | Bangladeshi BGE | 6 | 0 | 0 % | 6 | 100 % | [9] |
|  | Bangladeshi Garole | 6 | 0 | 0 % | 6 | 100 % | [9] |
|  | Changthangi | 15 | 0 | 0 % | 15 | 100 % | [9] |
|  | Garut | 8 | 2 | 25 % | 6 | 75 % | [9] |
|  | Indian Garole | 4 | 0 | 0 % | 4 | 100 % | [9] |
|  | Sumatra | 9 | 2 | 22 % | 7 | 78 % | [9] |
|  | **South Asia Total** | **48** | **4** | **8 %** | **44** | **92 %** |  |
| Central Europe | |  |  |  |  |  |  |
|  | Black Headed Mountain | 11 | 11 | 100 % | 0 | 0 % | [9] |
|  | Bunder Oberlander Sheep | 6 | 5 | 83 % | 1 | 17 % | [9] |
|  | East Friesian Brown | 15 | 0 | 0 % | 15 | 100 % | [9] |
|  | East Friesian White | 7 | 0 | 0 % | 7 | 100 % | [9] |
|  | Engadine Red Sheep | 10 | 0 | 0 % | 10 | 100 % | [9] |
|  | Swiss Black-Brown Mountain Sheep | 6 | 6 | 100 % | 0 | 0 % | [9] |
|  | Swiss Mirror Sheep | 10 | 9 | 90 % | 1 | 10 % | [9] |
|  | Swiss White Alpine Sheep | 8 | 8 | 100 % | 0 | 0 % | [9] |
|  | Valais BlacknoseSheep | 4 | 0 | 0 % | 4 | 100 % | [9] |
|  | Valais Red Sheep | 5 | 0 | 0 % | 5 | 100 % | [9] |
|  | Black-brown Milk sheep | 9 | 0 | 0 % | 9 | 100 % | [12] |
|  | Bentheim | 7 | 0 | 0 % | 7 | 100 % | [12] |
|  | Bavarian Forest sheep | 6 | 1 | 17 % | 5 | 83 % | [12] |
|  | Black-headed Mutton | 8 | 8 | 100 % | 0 | 0 % | [12] |
|  | Brown Mountain sheep | 5 | 0 | 0 % | 5 | 100 % | [12] |
|  | Coburg | 9 | 0 | 0 % | 9 | 100 % | [12] |
|  | Carynthian sheep | 10 | 9 | 90 % | 1 | 10 % | [12] |
|  | East Friesian Milk sheep | 7 | 0 | 0 % | 7 | 100 % | [12] |
|  | Forest sheep | 2 | 0 | 0 % | 2 | 100 % | [12] |
|  | German Gray Heath | 10 | 0 | 0 % | 10 | 100 % | [12] |
|  | GM German Merino | 4 | 2 | 50 % | 2 | 50 % | [12] |
|  | Leine | 8 | 5 | 63 % | 3 | 38 % | [12] |
|  | Merino Longwool | 5 | 0 | 0 % | 5 | 100 % | [12] |
|  | Mutton Merino | 9 | 1 | 11 % | 8 | 89 % | [12] |
|  | Pomeranian Coarsewool | 9 | 7 | 78 % | 2 | 22 % | [12] |
|  | Skudde | 9 | 0 | 0 % | 9 | 100 % | [12] |
|  | Tyrolean Mountain sheep | 11 | 0 | 0 % | 11 | 100 % | [12] |
|  | Tyrolean Stone sheep | 12 | 0 | 0 % | 12 | 100 % | [12] |
|  | White-horned Heath | 9 | 1 | 11 % | 8 | 89 % | [12] |
|  | White-headed Mutton | 6 | 6 | 100 % | 0 | 0 % | [12] |
|  | White Mountain sheep | 4 | 0 | 0 % | 4 | 100 % | [12] |
|  | White-polled Heath | 9 | 0 | 0 % | 9 | 100 % | [12] |
|  | **Central Europe Total** | **250** | **79** | **32** % | **171** | **68** % |  |
| North Europe | |  |  |  |  |  |  |
|  | Viena | 6 | 6 | 100 % | 0 | 0 % | [12] |
|  | Vepsia | 5 | 1 | 20 % | 4 | 80 % | [12] |
|  | Finnsheep | 73 | 56 | 77 % | 17 | 23 % | [9,12] |
|  | Kainuu Grey Sheep | 7 | 4 | 57 % | 3 | 43 % | [12] |
|  | ALD Aland sheep | 5 | 3 | 60 % | 2 | 40 % | [12] |
|  | ancient Finnish sheep | 3 | 3 | 100 % | 0 | 0 % | This study |
|  | Norwegian spaelsau | 15 | 5 | 33 % | 10 | 67 % | [9] |
|  | Spael coloured | 3 | 3 | 100 % | 0 | 0 % | [9] |
|  | Spael white | 26 | 19 | 73 % | 7 | 27 % | [9] |
|  | **North Europe Total** | **143** | **100** | **70** % | **43** | **30** % |  |
| North-West Europe | |  |  |  |  |  |  |
|  | Australian Poll Dorset | 108 | 108 | 100 % | 0 | 0 % | [9] |
|  | Border Leicester | 48 | 48 | 100 % | 0 | 0 % | [9] |
|  | Boreray | 3 | 3 | 100 % | 0 | 0 % | [9] |
|  | Dorset Horn | 4 | 1 | 25 % | 3 | 75 % | [9] |
|  | Galway | 4 | 4 | 100 % | 0 | 0 % | [9] |
|  | Irish Suffolk | 39 | 39 | 100 % | 0 | 0 % | [9] |
|  | Scotish Blackface | 56 | 0 | 0 % | 56 | 100 % | [9] |
|  | Soya | 25 | 0 | 0 % | 25 | 100 % | [9] |
|  | Black/white Suffolk | 116 | 113 | 97 % | 3 | 3 % | [9,12] |
|  | Coopworth | 23 | 16 | 70 % | 7 | 30 % | [9,12] |
|  | Border Leister | 1 | 1 | 100 % | 0 | 0 % | [12] |
|  | English Leister | 4 | 4 | 100 % | 0 | 0 % | [12] |
|  | Lincoln | 10 | 9 | 90 % | 1 | 10 % | [12] |
|  | PER Perendale | 1 | 0 | 0 % | 1 | 100 % | [12] |
|  | Polwarth | 3 | 0 | 0 % | 3 | 100 % | [12] |
|  | ROM Romney | 32 | 32 | 100 % | 0 | 0 % | [12] |
|  | Whitshire | 14 | 14 | 100 % | 0 | 0 % | [12] |
|  | **North-West Europe Total** | **491** | **392** | **80** % | **99** | **20** |  |
| South Europe | |  |  |  |  |  |  |
|  | Altamurana | 7 | 0 | 0 % | 7 | 100 % | [9] |
|  | Castellana | 18 | 0 | 0 % | 18 | 100 % | [9] |
|  | Churra | 108 | 0 | 0 % | 108 | 100 % | [9] |
|  | Leccese | 9 | 0 | 0 % | 9 | 100 % | [9] |
|  | Meat Lacaune | 75 | 57 | 76 % | 18 | 24 % | [9] |
|  | Merino landschaf | 19 | 0 | 0 % | 19 | 100 % | [9] |
|  | Milk Lacaune | 97 | 46 | 47 % | 51 | 53 % | [9] |
|  | Ojalda | 14 | 1 | 7 % | 13 | 93 % | [9] |
|  | Rambouillet | 74 | 12 | 16 % | 62 | 84 % | [9] |
|  | Sardinian Ancestral Black | 5 | 0 | 0 % | 5 | 100 % | [9] |
|  | Rasa Aragonesa | 14 | 1 | 7 % | 13 | 93 % | [9,12] |
|  | Latxa | 12 | 0 | 0 % | 12 | 100 % | [12] |
|  | Manchega | 10 | 0 | 0 % | 10 | 100 % | [12] |
|  | Mouflon (ovis musimon) | 10 | 0 | 0 % | 10 | 100 % | [12] |
|  | **South Europe Total** | **472** | **117** | **25** % | **355** | **75** % |  |
| Near East |  |  |  |  |  |  |  |
|  | Afshari | 5 | 0 | 0 % | 5 | 100 % | [9] |
|  | Cyprus Fat Tail | 5 | 0 | 0 % | 5 | 100 % | [9] |
|  | Karakas | 10 | 2 | 20 % | 8 | 80 % | [9] |
|  | Moghani | 27 | 0 | 0 % | 27 | 100 % | [9] |
|  | Norduz | 10 | 0 | 0 % | 10 | 100 % | [9] |
|  | Qezel | 30 | 0 | 0 % | 30 | 100 % | [9] |
|  | Sakiz | 11 | 0 | 0 % | 11 | 100 % | [9] |
|  | Awassi | 10 | 0 | 0 % | 10 | 100 % | [12] |
|  | **Near East Total** | **108** | **2** | **2** % | **106** | **98** % |  |
|  |  |  |  |  |  |  |  |
| Caucasus |  |  |  |  |  |  |  |
|  | Carpathian Mountain sheep | 2 | 0 | 0 % | 2 | 100 % | [12] |
|  | Bozakh | 5 | 0 | 0 % | 5 | 100 % | [12] |
|  | Tsigai | 5 | 0 | 0 % | 5 | 100 % | [12] |
|  | **Caucasus total** | **12** | **0** | **0 %** | **12** | **100 %** |  |
| North America | |  |  |  |  |  |  |
|  | Bighorn sheep (Ovis canadensis) | 6 | 0 | 0 % | 6 | 100 % | [12] |
|  | Thinhorn – Dall’s (Ovis dalli dalli) | 19 | 0 | 0 % | 19 | 100 % | [12] |
|  | Thinhorn – Stone’s (Ovis dalli stonei) | 9 | 0 | 0 % | 9 | 100 % | [12] |
|  | **North America total** | **34** | **0** | **0 %** | **34** | **100 %** |  |

n is the number of rams tested.
