# Supplementary material for: Mitochondrial DNA and Y-chromosomal diversity in ancient populations of domestic sheep (Ovis aries) in Finland: comparison with contemporary sheep breeds
Source: Genet Sel Evol. 2013 Jan 22;45(1):2. doi: 10.1186/1297-9686-45-2 (PMC3558444; doi:10.1186/1297-9686-45-2)
Supplement: Additional file 5 — Figure S2. Title: Mitochondrial DNA haplotypes (H) identified in 26 ancient and 94 modern sheep. Description: The data provided represent alignment of 26 ancient and 94 modern sheep analysed in this study. SNP positions given are relative to the reference sequence [GenBank:NC001941]. [file 1297-9686-45-2-S5.doc]

| 1111111111111111111111111111111111111111111111111111 |  |
| --- | --- |
| 5555555566666666666666666666666666666666666666666666 |  |
| 9999999900000000000011111111122222233334444444444444 |  |
| 7888889900122344569902233344500111444590011234445679 |  |
| 8012450338902628566718902347629247403620904920143226 |  |
| T-TAACTCTGCATGTTACCAACTACTCTCTCTTCA-TGTGGGACATCCGTTT  C-.G....C..GCACC..TG.T........T..T.CC........C..A.-.  C-.G.......GCACC..TG.T.......CT..T.CC........C..A.-.  CA.G.......GCACC..TG.T........T..T.CC...A....C..A.-.  C-.G.......GCACC..TG.TC.......TA.T.CC........C..A.-.  C-.G.......GCACC..TG.T........T..T.CC........C..A.-.  C-.G.......GCACC..TGGT........T..T.CC........C..A.-.  C-.G.......GCACC.TTGGT........T..T.CC........C..A.-.  C-.G.....A.GCACC..TG.T........T..T.CC........C..A.-.  C-.G.......GCACC..TG.T........T..T.CC......T.C..A.-.  C-.G.......GCACC..TG.T........T..T.CC........C.TA.-.  C-.G.......GCACCG.TG.T........T..T.CC........C..A.-.  C-.G.......GCAC.G.TG.T........T..T.CC........C..A.-.  C-CG.......G.ACC..TG.T........T..T.CC........C..A.-.  C-.G.......GCACC..TG.T..T.....T..T.CC...........A.-.  .-...................T...........T.CC...........A.-.  .-...........A.......T...........TGCC.............-.  .-..G........A.......T.G.........TGCC.............-.  .-...................T.G.........T.CC...........A.-.  .-...................T.G..T......T.CC........C....-.  .-.........G.........T.............CC.............-.  .-......C..G...C.....T.............CC...........A.-C  .-...................T.............CC...........A.-.  .-...................T......T......CC...........A.-.  .-...................T..T..........CC........C....-.  .-...................T.............CC........C....-.  .-...................T..T..........CC.............-.  .-...................T.............CCA............-.  .-...................T.............CC.........T...-.  .-....C..............T.............CC.............-.  .-...................T..........C..CC.............-.  .-...................T...C.........CC.............-.  .-...................T.............CC.............-.  .-.......A...........T.............CC.............-.  .-...................T....T........CC..........T..-.  .-...................T.............CC..........T..-.  .-C..................T.............CC..........T....  .-.......A...........T.............CC..........T..-.  .-...................T.............CC.C...........-.  C-...................T.............CC.............-.  .-...................T.............CC....A........-.  .-.............C.....T.............CC....A........-.  .-...................T.............CC..A..........-.  .-...................T.............CC..A.A........-.  .-.....T.............T.............CC....A..........  .-.....T.............T.............CC.....G.......-.  .-...........A.......T.............CC......TG.....-.  .-...........A.......T.............CC......T......-.  .-...........A.......T.............CC.............-.  .-...........AC......T.............CC.............-.  .-.....T.....A.......T.............CC.............-.  .-.....T.....A.......T.....C.......CC.............-.  .-...T...............T.............CC.............-.  .-...T...............T.............CC.............-C  .-........T....C.....T..T........T.CC............C-.  .-.............C.....T...........T.CC.............-.  .-.G..............T..T...........T.CC...........A.-. | NC001941  H01  H02  H03  H04  H05  H06  H07  H08  H09  H10  H11  H12  H13  H14  H15  H16  H17  H18  H19  H20  H21  H22  H23  H24  H25  H26  H27  H28  H29  H30  H31  H32  H33  H34  H35  H36  H37  H38  H39  H40  H41  H42  H43  H44  H45  H46  H47  H48  H49  H50  H51  H52  H53  H54  H55  H56 |

**Additional file 5, Figure S2**

Mitochondrial DNA haplotypes (H) identified in 26 ancient and 94 modern sheep.

Vertical numbers indicate the SNP positions relative to the reference sequence [GenBank:NC001941]. Only the variable sites are indicated. A dot (.) indicates a nucleotide similar to that in the reference sequence. A dash (-) indicates the position of an indel. Among the 56 haplotypes numbered H01 to H56, H1 to H14 belong to sheep haplogroup A and H15 to H56 to haplogroup B.
