# Supplementary material for: Mitochondrial DNA and Y-chromosomal diversity in ancient populations of domestic sheep (Ovis aries) in Finland: comparison with contemporary sheep breeds
Source: Genet Sel Evol. 2013 Jan 22;45(1):2. doi: 10.1186/1297-9686-45-2 (PMC3558444; doi:10.1186/1297-9686-45-2)
Supplement: Additional file 6 — Figure S3 and S4. Title: Median-joining network and mismatch distribution of the 56 mitochondrial haplotypes. Description: Figure S3 shows the median-joining network (ε = 0) with molecular relationships between 56 haplotypes which cluster into two major ovine haplogroups: haplogroup A (H01-H14, on the left) and haplogroup B (H15-H56, on the right). Figure S4 presents the mismatch distribution of 120 modern and ancient domestic sheep indicating the existence of two divergent haplogroups in the data. [file 1297-9686-45-2-S6.docx]

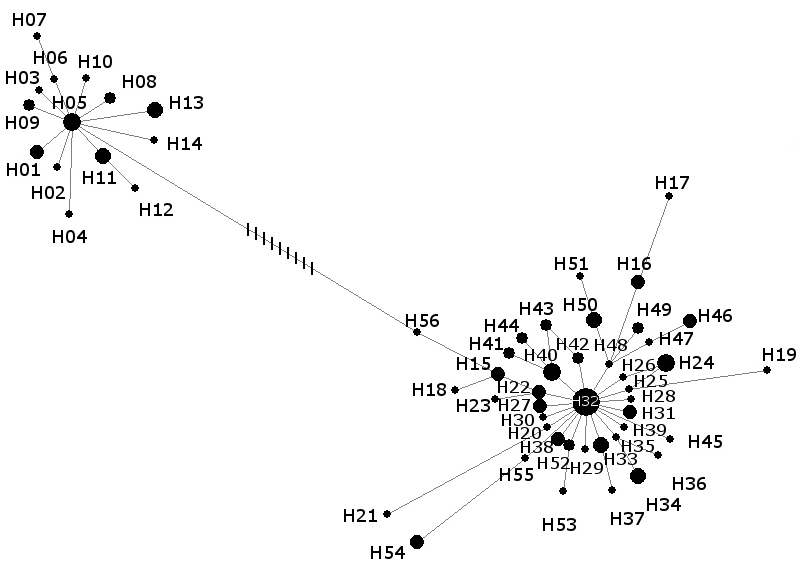


### Additional file 6

Figure S3 Median-joining network (ε = 0) with molecular relationships between 56 haplotypes which cluster into two major ovine haplogroups: haplogroup A (H01-H14, on the left) and haplogroup B (H15-H56, on the right).


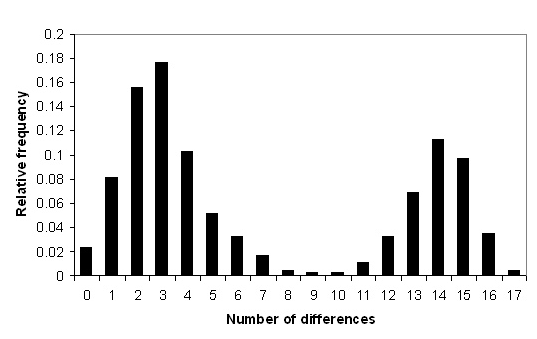


### Additional file 6

Figure S4 Mismatch distribution of 120 modern and ancient domestic sheep indicating the existence of two divergent haplogroups in the data.
